# Associations Between Apparent Diffusion Coefficient Values of Breast Tumors Presenting as Non-Mass Enhancement and Ki-67 Index

**DOI:** 10.3390/cancers18213560

**Published:** 2026-11-05

**Authors:** Iva Perić, Boris Brkljačić, Tade Tadić, Kristian Jerković, Krešimir Dolić, Martina Luetić, Monika Bilandžić, Matija Borić, Marija Ćavar

**Affiliations:** 1 Department of Diagnostic and Interventional Radiology, University Hospital of Split, Spinčićeva 1, 21000 Split, Croatia; 2 Department of Diagnostic and Interventional Radiology, University Hospital of Dubrava, Avenija Gojka Šuška 6, 10000 Zagreb, Croatia; 3 Department of Pathology, University Hospital of Split, Spinčićeva 1, 21000 Split, Croatia; 4 Independent Researcher, 21000 Split, Croatia; 5 Department of Abdominal Surgery, University Hospital of Split, Spinčićeva 1, 21000 Split, Croatia

**Keywords:** breast, breast cancer, MR diffusion-weighted imaging, NME, ADC values, Ki-67

## Abstract

Objectives: This study aimed to investigate whether apparent diffusion coefficient (ADC) values of malignant breast lesions presenting as non-mass enhancement (NME) are associated with the Ki-67 proliferation index. Materials and Methods: This retrospective, single-center study included 70 patients with malignant breast lesions presenting as NME on MRI. Patients were divided into two groups according to their Ki-67 index; Group K_L_ (Ki-67 < 20%) and K_H_ (Ki-67 ≥ 20%). All examinations were performed on a 1.5T scanner. Two radiologists, blinded to histopathology, independently outlined the whole NME on every subtraction slice on which it was visible and transferred these regions of interest to the ADC maps; the mean ADC value was used for subsequent analysis. Results: Mean ADC values were lower in Group K_H_ than in Group K_L_ (0.897 ± 0.158 vs. 1.116 ± 0.155 × 10^3^ mm^2^/s, *p* < 0.001) and were inversely correlated with the Ki-67 index (Spearman’s ρ = −0.458, 95% CI −0.626 to −0.250, *p* < 0.001). A high Ki-67 index remained associated with lower ADC values after adjustment for molecular subtype (b = −0.228 × 10^−3^ mm^2^/s; *p* < 0.001). ROC curve analysis yielded an AUC of 0.833 (95% CI 0.725 to 0.912). The exploratory Youden threshold of 1.045 × 10^−3^ mm^2^/s yielded a sensitivity of 85.7% and a specificity of 66.7%. Bootstrap resampling confirmed that the AUC was internally stable (mean 0.832, 95% CI 0.728 to 0.918). Conclusions: In malignant NME, lower ADC values are associated with a higher Ki-67 index. The ADC may add noninvasive information on tumor proliferation but cannot replace histopathology. The proposed threshold is exploratory and needs external validation before clinical use.

## Simple Summary

Breast cancer can appear as non-mass enhancement (NME) on magnetic resonance imaging (MRI). The Ki-67 index, measured on histopathology, is a routine marker of tumor proliferation. This study investigated the relationship between apparent diffusion coefficient (ADC) values and the Ki-67 index in 70 patients with malignant NME. Patients were divided into two groups according to their Ki-67 index, Group K_L_ (Ki-67 < 20%) and K_H_ (Ki-67 ≥ 20%). ADC values were lower in tumors with a higher Ki-67 index, suggesting that ADC may partly reflect tumor proliferation.

## 1. Introduction

Breast cancer remains a major global health concern, with its incidence continuing to rise [1,2,3]. In recent decades, treatment has advanced considerably, and the multimodal approach has notably reduced mortality and prolonged patients’ lives [3,4,5,6]. A well-established treatment method is neoadjuvant therapy, which includes various oncological modalities. Patient selection for neoadjuvant therapy follows established guidelines and considers tumor size, nodal status, hormone receptor and HER2 status, and markers of tumor proliferation [7,8,9].

One of the radiological presentations of breast tumors is pathological malignant enhancement visible on contrast imaging (e.g., MRI or CEM) [10], which does not meet the criteria for classification as a mass and is therefore characterized as non-mass enhancement (NME). In such cases, tumor size is harder to assess than in lesions with a well-defined volume [11,12]. Histopathological analysis of biopsy specimens characterizes these tumors, including their Ki-67 proliferation index [13].

Breast MRI allows for both morphological and quantitative lesion assessment, and diffusion-weighted imaging (DWI) with apparent diffusion coefficient (ADC) maps is well established [14,15,16,17,18,19,20]. In our previous research [21], we demonstrated that measuring the average ADC value of the entire volume of NME can be useful for the initial noninvasive assessment of whether the MRI enhancement is benign or malignant. That study included both benign and malignant NME and did not assess tumor proliferation.

The aim of this study was to evaluate whether the ADC value of malignant lesions presenting as NME, measured using the average of the entire NME volume, is associated with the level of tumor proliferation, as measured by the Ki-67 index.

## 2. Materials and Methods

### 2.1. Patients

This single-center study was approved by the Ethics Committee at the University Hospital of Split (Class: 520-03/23-01/37; Reference no.: 2181-147/01/06LJ.Z.-26-04, 15 October 2026), with formal consent waived due to its retrospective design.

We retrospectively searched the local hospital database for patients who underwent breast MRI between January 2020 and June 2026. Of the 70 patients, 25 (35.7%) were also included in our previous study [21], which addressed the differentiation of malignant and benign NME using ADC values. The present study tests a different hypothesis: it includes only invasive malignant NME and examines the association between ADC values and the Ki-67 index. None of the analyses reported here were performed in the previous study. The inclusion criteria for this study were histopathologically confirmed invasive malignancies presenting as NME in patients older than 18 years. Patients were excluded if the DWI or subtraction images were of insufficient quality for analysis. All consecutive patients who met the eligibility criteria during the study period were included.

Based on our previous study [21], a sample size of 28 patients per group was required to achieve statistical power of 80% (α 0.05 and β 0.2) to detect differences of 0.200 × 10^−3^ mm^2^/s in ADC values between Ki-67 groups. The final cohort included 70 patients.

Patients were divided into two groups according to the Ki-67 index [13]: Group K_L_ (Ki-67 < 20%) and Group K_H_ (Ki-67 ≥ 20%).

### 2.2. MR Scanning Protocol

All patients underwent MRI using a 1.5T MR scanner (Aera, Siemens Healthineers, Erlangen, Germany). Patients were scanned prone in a dedicated breast coil using a standardized protocol. Following the pre-contrast scan, a gadolinium-based contrast agent was administered.

The imaging protocol consisted of the following sequences: (1) axial turbo spin echo inversion recovery sequence with magnitude reconstruction or TIRM (TR/TE/TI values of 5200/57/170 ms, FOV of 347 mm × 370 mm, and a slice thickness of 4 mm); (2) DWI sequence using spectral attenuated inversion recovery or SPAIR (TR/TE, 5000/73 ms, FOV 176 mm × 400 mm; slice thickness 4.5 mm), with three b values of 50, 400, and 800 s/mm^2^; (3) axial T1 fat-suppressed weighted images for dynamic imaging (TR/TE values of 5.3/2.4 ms, FOV of 375 mm × 300 mm, and a slice thickness of 1.6 mm); (4) subtracted images generated automatically by the operating system.

Besides the pre-contrast acquisition of T1-weighted, T2-weighted, and diffusion sequences, six post-contrast images were acquired. The first post-contrast image was obtained two minutes after contrast administration, and subtraction images were then generated automatically. The total examination time was approximately 15 min.

### 2.3. Image Evaluation

Two radiologists experienced in breast MRI independently reviewed all examinations, using the MR Breast Workflow on Syngo.via (Siemens Healthineers, Forchheim, Germany). Both readers were blinded to each other’s assessments and to the histopathological findings, including tumor type and Ki-67 index.

The image review included T1-weighted images, TIRM sequences, DWI, ADC maps, and a third post-contrast subtraction sequence.

For ADC measurement, each reader manually outlined the whole NME on the third subtraction image, correlating it with pre-contrast sequences, carefully avoiding areas of necrosis, and obtaining the whole-NME region of interest (ROI). The ROI was then transferred to the corresponding ADC map using the Syngo.via MR Breast Workflow. This was repeated on every subtraction slice that had a corresponding ADC slice. For each reader, the mean ADC value was calculated across all delineated slices, with each slice contributing equally. Inter-reader agreement was then assessed.

### 2.4. Histopathology

Breast core needle biopsy was performed according to a standardized protocol, with a minimum of three tissue cores obtained per lesion. Specimens were immediately immersed in 10% neutral buffered formalin after collection and submitted to the Department of Pathology on the following day. The specimens were fixed for 24–48 h, after which gross examination was performed by an experienced breast pathologist. Tissue samples were dehydrated through graded alcohols, cleared with xylene, and infiltrated with paraffin using an automated tissue processor (Thermo Shandon, Runcorn, UK). The processed tissue was embedded in paraffin, and 4 µm thick sections were cut using a microtome. The sections were then transferred to a hot water bath, mounted on glass slides, and stained with hematoxylin and eosin (H&E) using an automated staining system (Ventana HE 600, Roche Diagnostics, Tucson, AZ, USA).

The histopathological slides were evaluated by a breast pathologist with 10 years of experience—blinded to MRI reports and ADC values—using an Olympus BX43 light microscope (Olympus, Tokyo, Japan). Immunohistochemical staining for Ki-67 was carried out using the Ventana Benchmark Ultra Plus System (Roche Diagnostics, Tucson, AZ, USA) and a Ki-67 antibody (Dako, Agilent Technologies, Denmark) at a dilution of 1:100. The Ki-67 index was evaluated using a light microscope (Olympus BX61) equipped with a cooled digital camera (DP71, Olympus) and CellSens Standard 1.9 imaging software (Olympus). The Ki-67 index was determined using the global average method, whereby representative tumor areas were selected throughout the specimen while avoiding overrepresentation of highly proliferative regions. At least 500 tumor cell nuclei were counted for each case, and the Ki-67 index was calculated as the percentage of positively stained nuclei among the total number of evaluated tumor cell nuclei.

No universally accepted Ki-67 cutoff exists, and reported thresholds vary between institutions [13,22,23,24]. We applied the 20% threshold used routinely in our institution. This threshold matches the cutoff proposed in the 2013 St. Gallen consensus [25], although the interpretation of Ki-67 remains context-dependent and no single threshold is universally accepted.

### 2.5. Statistical Analysis

Statistical analyses were performed using the MedCalc^®^ statistical software, version 23.7.1 (MedCalc Software Ltd., Ostend, Belgium; https://www.medcalc.org, accessed on October 2026), with *p* < 0.05 considered statistically significant.

The dependent variable was the mean ADC value of the whole NME volume, and the grouping variable was the Ki-67 category (Group K_L_ and Group K_H_). For each lesion, the mean of whole-NME ADC values obtained by two readers was used in all subsequent analyses. Because ADC measurement in NME is not standardized and neither reader could serve as a reference, averaging the two measurements was intended to reduce reader-related error. Continuous data are presented as mean ± standard deviation and categorical data as counts and percentages. The comparison of ADC values between Ki-67 groups was defined as the primary analysis and was the basis of the a priori sample size calculation; multivariable regression and ROC analyses were considered secondary and exploratory.

The normality of the ADC values was assessed using the Shapiro–Wilk test. As ADC values were normally distributed, they were compared between Group K_L_ and Group K_H_ using Student’s *t*-test. The association between mean ADC values and the Ki-67 index treated as a continuous variable was assessed using Spearman’s rank correlation coefficient, reported with its 95% confidence interval. NME distribution and internal enhancement pattern were compared using Pearson’s chi-square test. Multivariable linear regression analysis was performed with ADC as the dependent variable and molecular subtype (reference category: HR+/Her2-) and Ki-67 group (reference group: K_L_) as independent variables. Regression coefficients (b) are reported with 95% confidence intervals, together with the coefficient of determination for the model. Normality of the residuals was assessed using the Kolmogorov–Smirnov test with Lilliefors correction. Owing to the small number of HR-/Her2+ tumors (n = 3), this subgroup was retained in the model for completeness, but no inference was drawn from its estimate. A sensitivity analysis was performed excluding HR-/Her2+ tumors. Histological grade was not included in the multivariable analysis.

Inter-reader agreement for mean ADC measurements was assessed using the intraclass correlation coefficient (ICC, absolute agreement, single measure) with its 95% confidence interval. Bland–Altman analysis was used to estimate the mean bias and the 95% limits of agreement between readers.

As an exploratory analysis, the diagnostic performance of ADC values in discriminating Group K_L_ from Group K_H_ was assessed by receiver operating characteristic (ROC) curve analysis with calculation of the area under the curve (AUC) and its 95% confidence interval. The optimal ADC threshold was determined using the Youden index, and an additional threshold corresponding to 90% specificity was identified. For each threshold, sensitivity, specificity, positive predictive value, negative predictive value and overall accuracy were calculated; predictive values reflect the prevalence of high Ki-67 observed in our cohort.

For internal validation, the stability of the ROC-derived estimates was tested by bootstrap resampling (1000 iterations). In each bootstrap sample, the ROC analysis was repeated and the optimal threshold (Youden index), AUC, sensitivity, and specificity were estimated; results are reported as means with 95% percentile intervals. Bootstrap analyses were performed in R (version 4.6.1; R Foundation for Statistical Computing, Vienna, Austria) using the pROC package (version 1.19.1).

## 3. Results

The study included 70 patients with malignant NME (mean age = 56 ± 13.5 years). Of the 70 malignant lesions, 42 (60%) had Ki-67 values below 20%, and 28 (40%) had Ki-67 index of 20% or higher. Histologically, 41 (58.6%) lesions were invasive ductal carcinoma, and 29 (41.4%) were invasive lobular carcinomas. Most lesions were HR+/Her2- (54/70, 77.1%) followed by HR+/Her2+ (13/70, 18.6%) and HR-/Her2+ (3/70, 4.3%). MRI morphological features are shown in Table 1.

Inter-reader agreement for mean ADC measurements was good, with an ICC value of 0.84 (95% CI 0.74–0.90).

**Figure 1:**
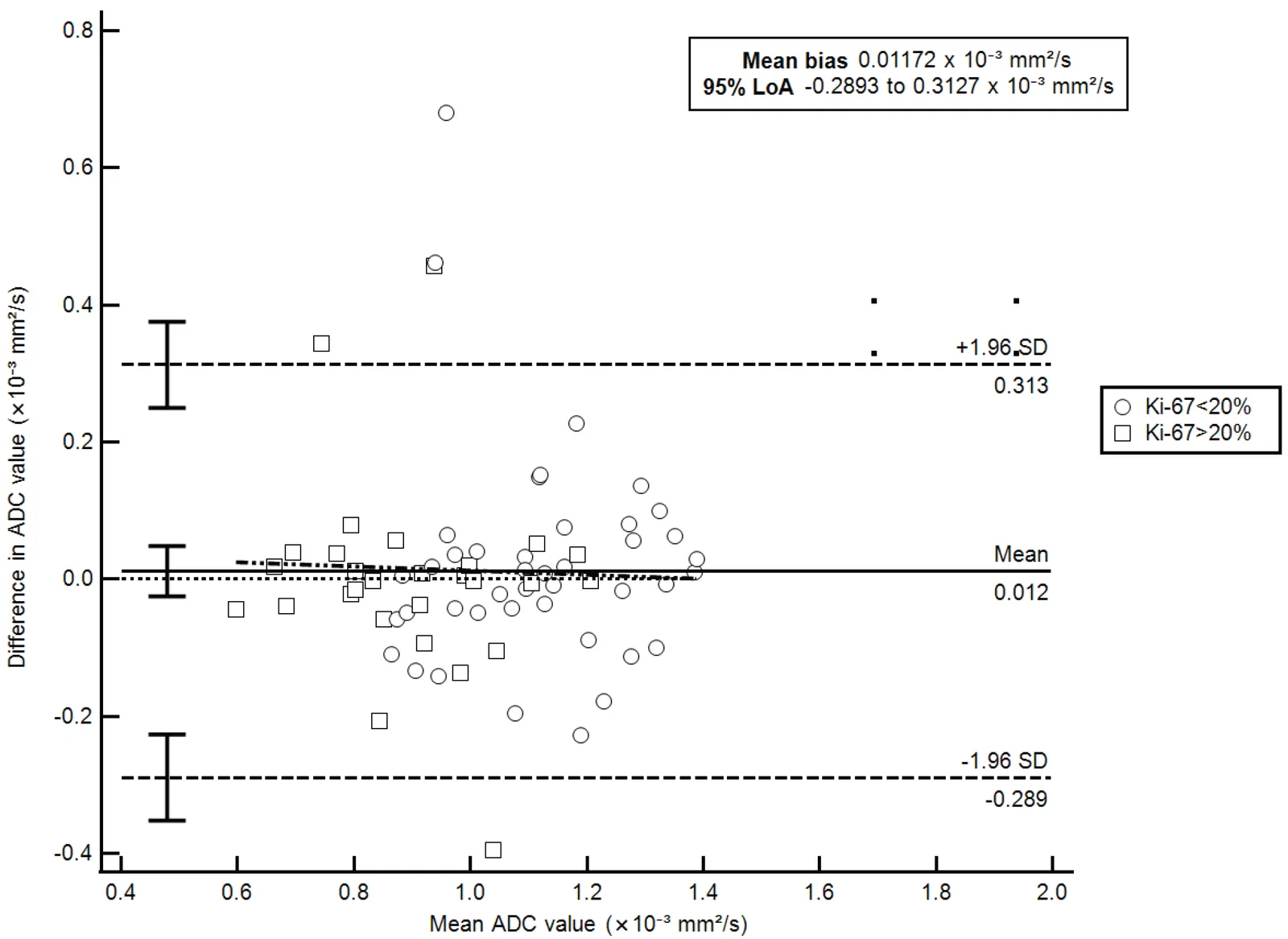
A Bland–Altman plot of inter-reader agreement for mean ADC measurements. The mean bias was 0.01172 × 10^−3^ mm^2^/s, and the 95% limits of agreement ranged from −0.2893 to 0.3127 × 10^−3^ mm^2^/s. The dashed lines represent the 95% limits of agreement, and the solid line represents the mean bias.

Agreement between the two readers was further demonstrated using Bland–Altman analyses (Figure 1).

**Figure 2:**
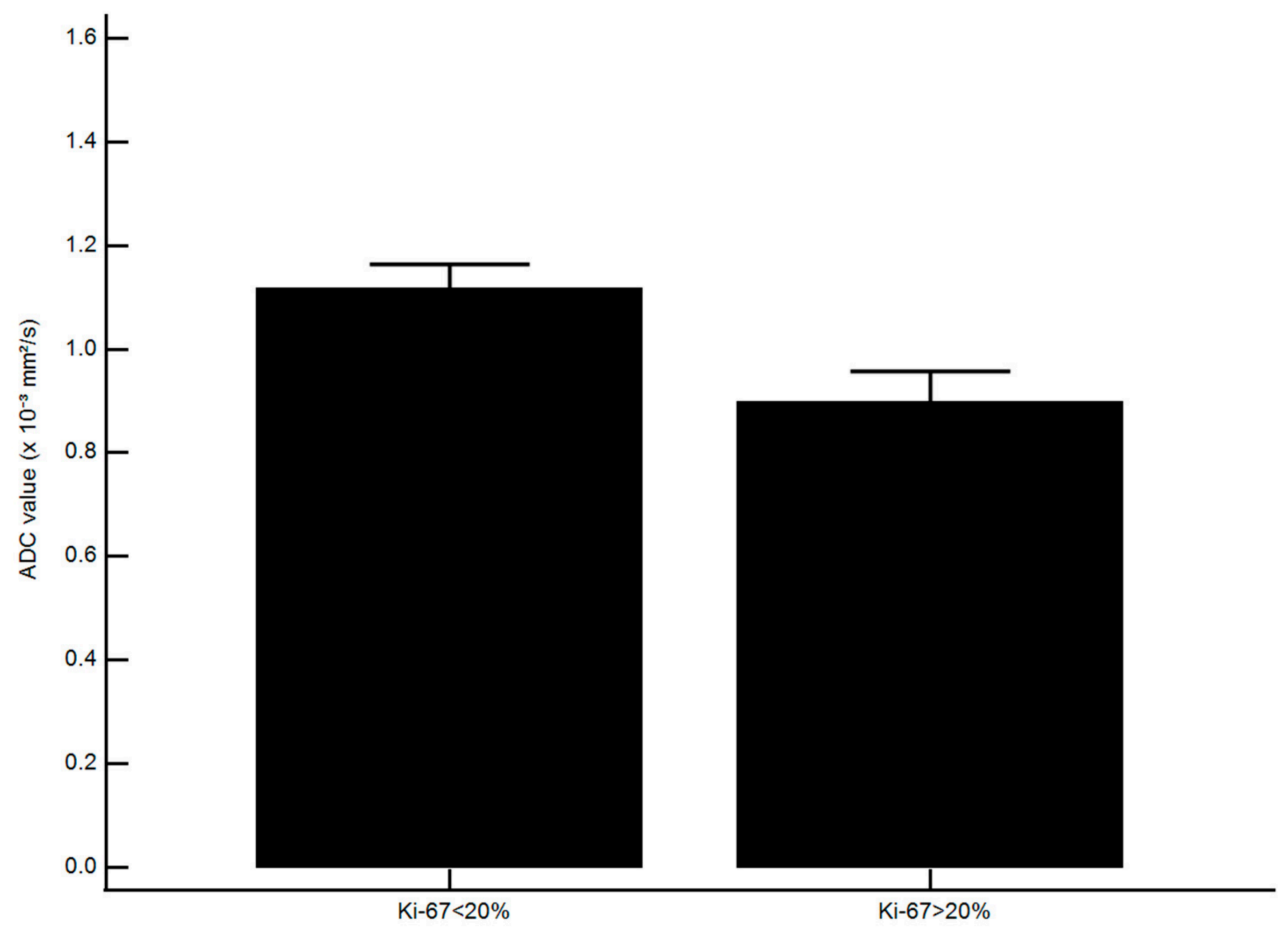
Bar graph showing mean ADC^a^ values in both groups, with SD^b^ as error bars. ^a^ ADC—apparent diffusion coefficient; ^b^ SD—standard deviation.

The mean ADC value in Group K_L_ (1.116 ± 0.155 × 10^−3^ mm^2^/s) was higher than in Group K_H_ (0.897 ± 0.158 × 10^−3^ mm^2^/s; mean difference −0.219 × 10^−3^ mm^2^/s, 95% CI −0.295 to −0.143, *p* < 0.001) (Figure 2).

**Figure 3:**
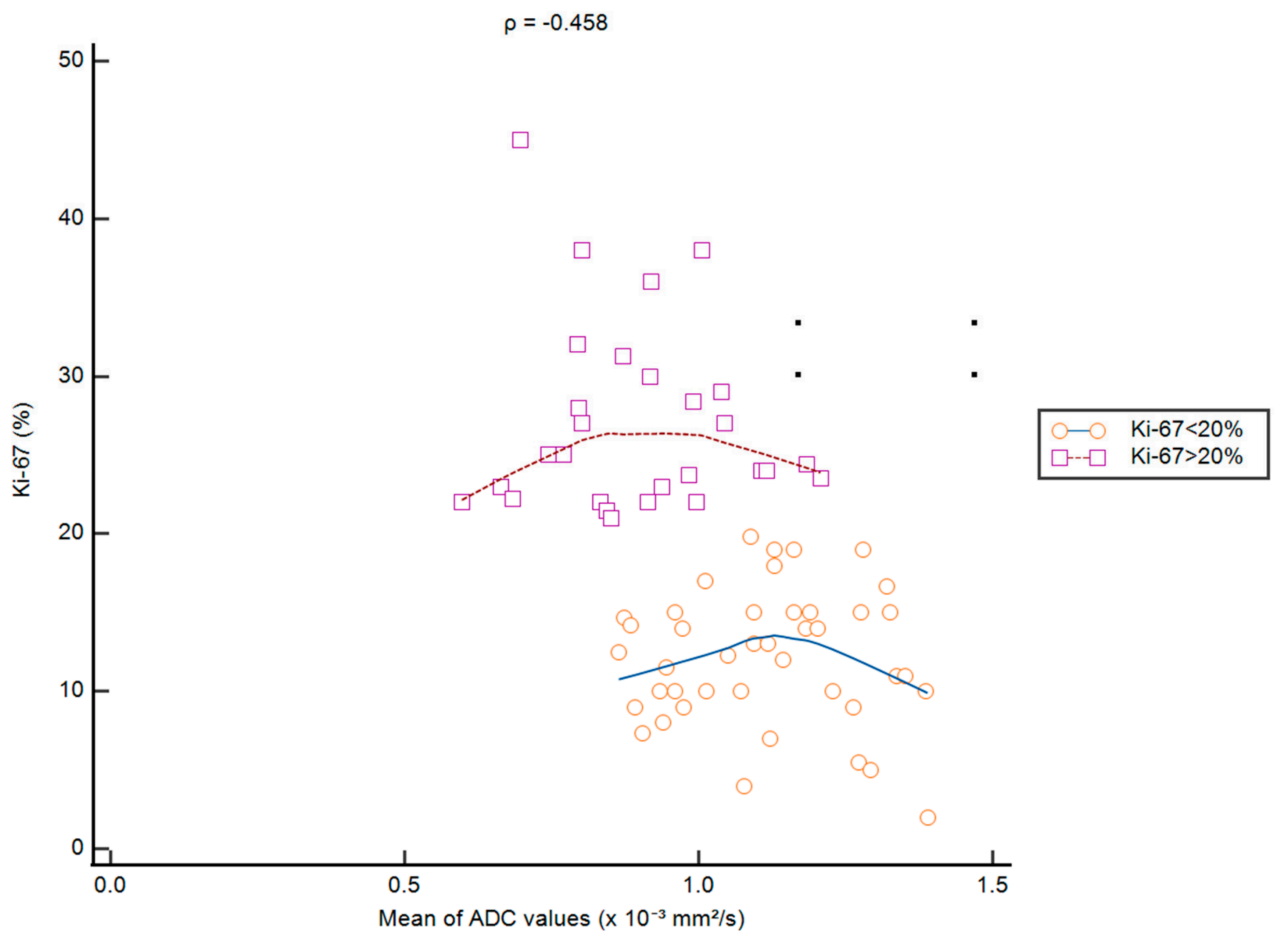
A scatter plot of mean ADC values against the Ki-67 index. Each point represents an individual lesion, with the Ki-67 index plotted as an exact percentage. Spearman’s ρ = −0.458 (*p* < 0.001).

Mean ADC values were inversely correlated with the Ki-67 index treated as a continuous variable (Spearman’s rank correlation coefficient ρ = −0.458, 95% CI −0.626 to −0.250, *p* < 0.001). The distribution of individual lesions is shown in Figure 3.

In the exploratory multivariable analysis, the model was statistically significant (F = 13.07, *p* < 0.001; R^2^ = 0.37). High Ki-67 was independently associated with lower ADC values (b = −0.228 × 10^−3^ mm^2^/s, 95% CI −0.305 to −0.150, *p* < 0.001). Molecular subtype was not significantly associated with ADC after adjustment for Ki-67 (HR+/Her2+: b = 0.083 × 10^−3^ mm^2^/s, 95% CI −0.011 to 0.178, *p* = 0.083). With only three HR-/Her2+ tumors, the estimate for this subtype was imprecise (b = 0.137, 95% CI −0.050 to 0.324). The residuals were normally distributed (Kolmogorov–Smirnov test, *p* > 0.10).

In a sensitivity analysis excluding the three HR-/Her2+ tumors (n = 67), the results were unchanged: high Ki-67 remained independently associated with lower ADC values (b = −0.228 × 10^−3^ mm^2^/s, 95% CI −0.305 to −0.150, *p* < 0.001), whereas HR+/Her2+ status was not (b = 0.083 × 10^−3^ mm^2^/s, 95% CI −0.011 to 0.178, *p* = 0.084; R^2^ = 0.38).

**Figure 4:**
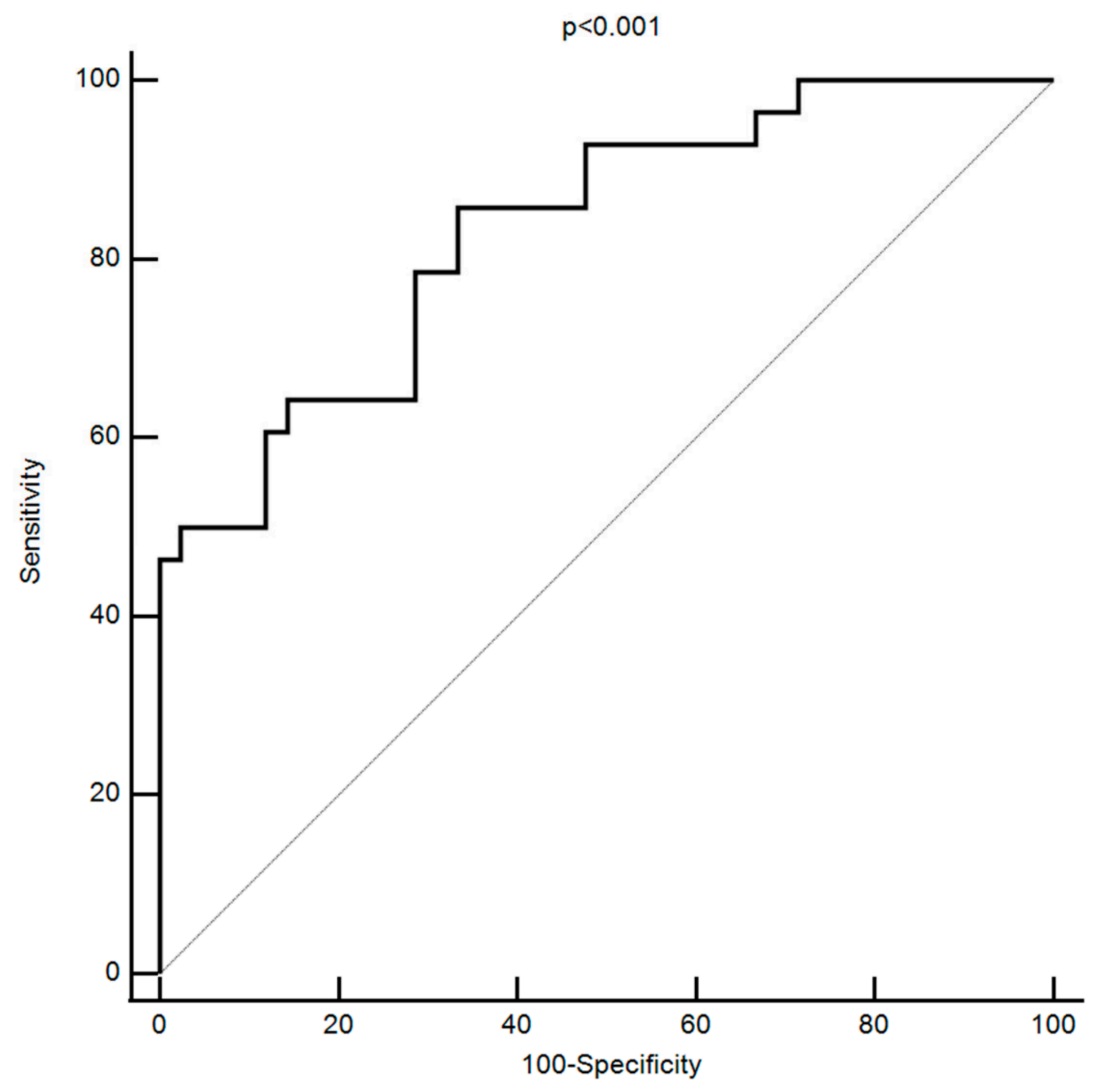
Receiver operating characteristic curve for NME^a^ for the whole-NME ADC in discriminating high from low Ki-67 (AUC 0.833). ^a^ NME—non-mass enhancement. The gray line is standard for ROC curve diagram.

Exploratory ROC curve analysis was performed to evaluate the diagnostic performance of ADC values and to identify candidate threshold values (Figure 4). The AUC was 0.833 (95% CI 0.725–0.912, *p* < 0.001). The threshold providing the best balance between sensitivity and specificity was 1.045 × 10^−3^ mm^2^/s, yielding a sensitivity of 85.7% (95% CI 67.3–96.0), specificity of 66.7% (95% CI 50.5–80.4), PPV of 63.2% (95% CI 46.0–78.2), NPV of 87.5% (95% CI 71.0–96.5), and accuracy of 74.3% (95% CI 62.4–84.0). A threshold of 0.894 × 10^−3^ mm^2^/s yielded 90% specificity, with a sensitivity of 50%. These threshold values were derived from the present study population and should be considered exploratory.

On bootstrap resampling (1000 resamples), the mean AUC was 0.832 (95% CI 0.728–0.918), close to the original estimate. The mean optimal ADC threshold was 0.985 × 10^−3^ mm^2^/s (95% CI 0.854–1.118 × 10^−3^ mm^2^/s), with a mean sensitivity of 76.6% (95% CI 46.4–100%) and mean specificity of 80.6% (95% CI 54.0–100%).

## 4. Discussion

Our results showed a negative association between ADC values and the Ki-67 proliferative index in malignant tumors presenting as NME on breast MRI, with lower ADC values being associated with higher proliferative activity. The correlation was moderate and held across the full Ki-67 range, not only at the 20% cutoff used for group comparison.

These findings are consistent with most previously published studies that reported a similar inverse correlation [18,26,27]. However, those studies mostly examined mass lesions [26,28], and few have addressed Ki-67 in NME [29,30]. Wang et al. [30] reported that the Ki-67 index was associated with the morphological subtype of NME, with “clustered ring enhancement” correlating positively with a higher Ki-67 index, but they did not assess ADC values directly and did not include the remaining NME subtypes.

Nie et al. [31] reported the opposite, a positive correlation between ADC values and the Ki-67 index in NME. The authors acknowledged that this contradicted earlier work and attributed the discrepancy to their exclusive focus on lesions presenting as NME. Differences in ADC measurement methods may also explain this discrepancy. Because NME does not form a well-defined mass, no standardized approach to ADC measurement exists for such cases. In our previous study [21], whole-NME volumetric measurement with calculation of the mean ADC value allowed for assessment of diffusion in NME. This approach may be particularly suited to infiltrative lesions with periductal and stromal growth patterns.

At the cellular level, malignant tissue differs from normal tissue partly in cellularity [32], and increased cell density with a reduced extracellular volume fraction restricts the diffusion of water molecules. Tumors with higher proliferative activity would therefore be expected to show lower ADC values, as we observed. The association was not explained by molecular subtype: in the multivariable analysis, high Ki-67 remained independently associated with lower ADC values after adjustment for HR/Her2 status. Although Ki-67 group explained most of the model variance, this does not mean that proliferative activity causally determines ADC values.

In our cohort, the candidate Youden-derived threshold of 1.045 × 10^−3^ mm^2^/s favored sensitivity (85.7%) and yielded a high negative predictive value (87.5%). In the bootstrap analysis, the mean AUC closely matched the original estimate. This supports the internal stability of the discriminatory ability of the ADC, but it does not establish the stability or generalizability of the proposed threshold, which varied considerably across resamples (95% CI 0.854–1.118 × 10^−3^ mm^2^/s). The ADC may provide additional information about tumor proliferation but does not directly measure tumor aggressiveness or replace histopathological assessment.

This study has several limitations. First, the Ki-67 index was dichotomized at 20% for the group comparison and the ROC analysis. Dichotomization discards information and makes results dependent on the chosen threshold. For this reason, the Ki-67 index was additionally analyzed as a continuous variable, and the inverse correlation with the ADC was present across its full range (Spearman’s ρ = −0.458), indicating that the findings are not an artifact of the chosen cutoff. The ADC thresholds were derived from a relatively small, single-center, retrospective cohort, and selection bias cannot be excluded. Bootstrap resampling showed stable discrimination within the dataset, but the optimal threshold varied with sample composition. The proposed thresholds were derived and internally evaluated in the same cohort; bootstrap resampling quantifies their uncertainty but does not replace validation in an independent population. These exploratory thresholds require validation in larger, independent prospective cohorts before they can be considered for clinical use. Although 70 patients with malignant NME is a modest sample, this remains one of the few studies to examine tumors presenting exclusively as NME on breast MRI in relation to the Ki-67 index.

Second, the ADC was measured manually. Our previous work showed that inter-reader and intra-reader variability were acceptable [21,33], but because ADC measurement in NME has not yet been standardized, the approach requires validation in larger prospective multicenter studies. Inter-reader agreement was good (ICC 0.84) with negligible mean bias, indicating no systematic difference between readers. However, the Bland–Altman limits of agreement were relatively wide, so lesions with ADC values close to a threshold could occasionally be classified differently by different readers. Averaging the two readers’ measurements may reduce this variability, but caution is needed when applying the thresholds to individual patients. The cohort partly overlaps with that of our previous study [21], although the present work addressed a different research question and included analyses not performed before.

Third, there was no direct spatial correspondence between the ADC measurements and the histopathological assessment of Ki-67. ADC values were obtained from the entire NME volume, whereas the Ki-67 index was determined on core needle biopsy specimens, which sample only a limited portion of the lesion. Although the use of a whole-NME ROI and the global average method for Ki-67 assessment was intended to reduce spatial mismatch, it does not ensure that the imaged and sampled tissue regions correspond. The observed relationship should therefore be interpreted as an association between lesion-level ADC values and the biopsy-derived Ki-67 index, rather than as a direct spatial correlation.

Fourth, Ki-67 is closely related to the molecular phenotype of breast cancer, as it is one component of the surrogate immunohistochemical classification. The association with the ADC may therefore partly reflect differences in molecular subtype rather than proliferation alone. Although Ki-67 remained associated with the ADC after adjustment for HR/Her2 status, the Her2-positive subgroups were small, no triple-negative tumors were included, and histological grade was not included in the multivariable analysis; residual confounding by these and other unmeasured tumor characteristics therefore cannot be excluded. Because most tumors were HR+/Her2-, the findings may not apply to Her2-enriched or triple-negative tumors.

Finally, the study was powered only for the primary comparison of ADC values between Ki-67 groups, for which the target sample size was reached; all other analyses were not powered a priori and should be regarded as exploratory.

## 5. Conclusions

In malignant breast tumors presenting as NME on MRI, lower ADC values were associated with a higher Ki-67 index. ADC measurements may provide additional noninvasive information on tumor proliferation in NME but cannot replace histopathology. As the cohort consisted predominantly of HR+/Her2- tumors, these findings may not apply to all molecular subtypes. The ADC thresholds derived in this retrospective single-center cohort are exploratory, are not suitable for routine clinical decision-making, and need prospective, multicenter validation.

## Figures and Tables

**Table 1:** NME^a^ and MR imaging descriptors.

	Malignant Lesions (n = 70) (%)	Ki-67 Low	Ki-67 High	*p*
Distribution				0.969
Focal	21 (30)	12 (17.1)	9 (12.9)	
Linear	6 (8.6)	4 (5.7)	2 (2.9)	
Segmental	16 (22.9)	10 (14.3)	6 (8.6)	
Regional	18 (25.7)	10 (14.3)	8 (11.4)	
Diffuse	9 (12.9)	6 (8.6)	3 (4.3)	
Internal enhancement pattern				0.919
Homogeneous	4 (5.7)	3 (4.3)	1 (1.4)	
Heterogeneous	45 (64.3)	26 (37.1)	19 (27.1)	
Clumped	16 (22.9)	10 (14.3)	6 (8.6)	
Clustered-ring	5 (7.1)	3 (4.3)	2 (2.9)	

^a^ NME—non-mass enhancement.

## Data Availability

Data available on request due to restrictions (privacy reasons).

## Abbreviations

The following abbreviations are used in this manuscript:

MRI	Magnetic Resonance Imaging
CEM	Contrast-enhanced mammography
NME	Non-mass enhancement
DWI	Diffusion-weighted imaging
ADC	Apparent diffusion coefficient
ROI	Region of interest
ICC	Intraclass correlation coefficient
ROC	Receiver Operating Characteristic
AUC	Area under the curve
PPV	Positive predictive value
NPV	Negative predictive value
